# A rare case of a splenic hamartoma in a patient with a huge palpable abdominal mass: a case report

**DOI:** 10.1186/1752-1947-9-4

**Published:** 2015-01-28

**Authors:** Paraskevi Vlachou, Dimitris Fagkrezos, Anastasia Tzivelopoulou, Georgia Kyriakopoulou, Petros Maniatis, Charikleia Triantopoulou, John Papailiou

**Affiliations:** 1 Department of Radiology, Konstantopouleio General Hospital, Agias Olgas 3-5, 14233 Nea Ionia, Greece; 2 Computed Tomography Department, Konstantopouleio General Hospital, Agias Olgas 3-5, 14233 Nea Ionia, Greece

**Keywords:** CT, Hamartoma, MRI findings, Spleen, Splenic hemangioma, Splenoma

## Abstract

**Introduction:**

Splenic hamartoma is a primary benign tumor of the spleen, which is often found incidentally. Splenic hamartomas are very rare, with approximately 150 cases documented in the literature to date. They represent benign vascular proliferation. Histological findings consist of disorganized stroma and vascular channels of varying width, with or without lymphoid follicles.

**Case presentation:**

We present the case of a 39-year-old Greek woman, with no significant medical history, who was diagnosed incidentally with an enormous splenic hamartoma on computed tomography, finally confirmed by surgery and histopathology. Hamartomas are benign lesions, and it is important to differentiate them from malignancy.

**Conclusion:**

Hamartoma represents a rare vascular entity characterized by a cluster of differentiation 8-positive immunophenotype. It is usually asymptomatic but large hamartomas may present with symptoms such as hemopoetic disorders, which resolve after splenectomy. It is important for radiologists to be able to differentiate splenic hamartoma from malignant entities.

## Introduction

In terms of structure and function, the spleen consists of white and red pulp [1]. White pulp plays an important role in the immune system by producing lymphocytes, plasma cells and antibodies. Red pulp filters blood, by removing microorganisms, old red cells and several antigens. In young people, the spleen also has a hemopoetic role. The length of the spleen does not exceed 13cm in men and 12cm in women. Its weight is between 80g and 300g, with a median weight of about 150g.

Vascular neoplasms are the most common primary neoplasms of the spleen. Spleen hamartoma was described first in 1861 by Rokitansky [2], and has also been called splenoma or spleen within a spleen.

Benign splenic tumor-like masses are generally rare, with an incidence of seven cases in 100,000 autopsy specimens, and most are cysts or hemangiomas. Hamartomas are extremely rare, with an incidence of three cases in 200,000 splenectomies [3].

With the rapid improvement of imaging modalities, the detection of small and asymptomatic lesions is now easy, and a differential diagnosis can be achieved in a timely manner with high accuracy.

## Case presentation

A 39-year-old Greek woman, with no remarkable medical history, presented to the emergency room of our hospital with diffuse abdominal pain and a mass-like distention of the left side of her abdomen. No weight loss was reported.

On physical examination we found a big painless, palpable mass on the left side of her abdomen. She had normal vital signs and laboratory findings were normal, except for mild leukocytosis.

An abdominal ultrasound showed a huge, mostly hypoechoic, mass that was impossible to characterize. Its origin was also difficult to define.

**Figure 1:**
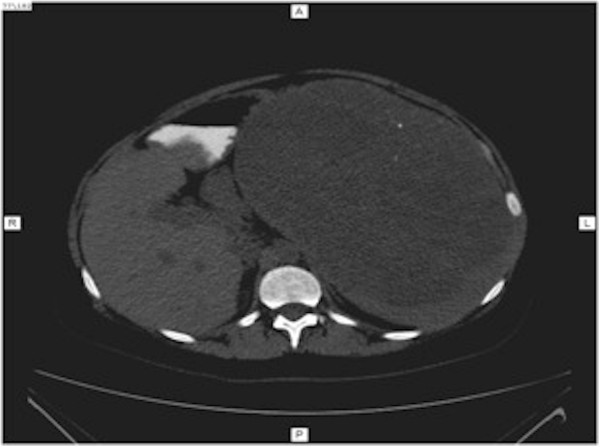
**Computed tomography imaging of splenic hamartoma before contrast injection.** Note the presence of two microcalcifications.

**Figure 2:**
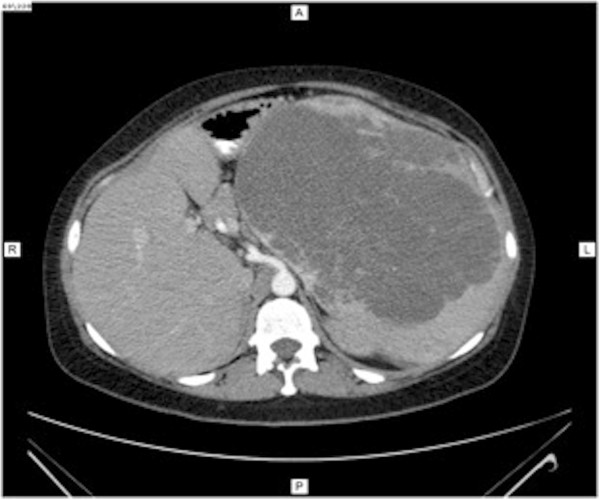
**Computed tomography imaging of the splenic mass in arterial phase.**

**Figure 3:**
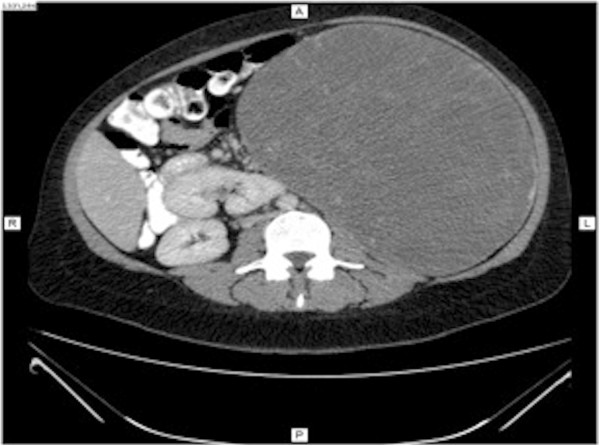
**Computed tomography imaging of the splenic mass in venous phase.**

Abdominal computed tomography (CT) was performed, and images before the administration of contrast material showed a slightly hypodense, well-circumscribed, encapsulated mass on her left abdominal side, measuring 23×17×23cm (Figure 1). This mass was compressing other abdominal organs to the right, with no obvious invasion. After contrast bolus injection, in the arterial phase, we noted heterogenous enhancement, with higher peripheral contrast media concentration (Figure 2). No vessel invasion was noticed. In the late venous phase, the mass became isodense. No cystic areas were observed but microcalcifications were noted (Figure 3).

**Figure 4:**
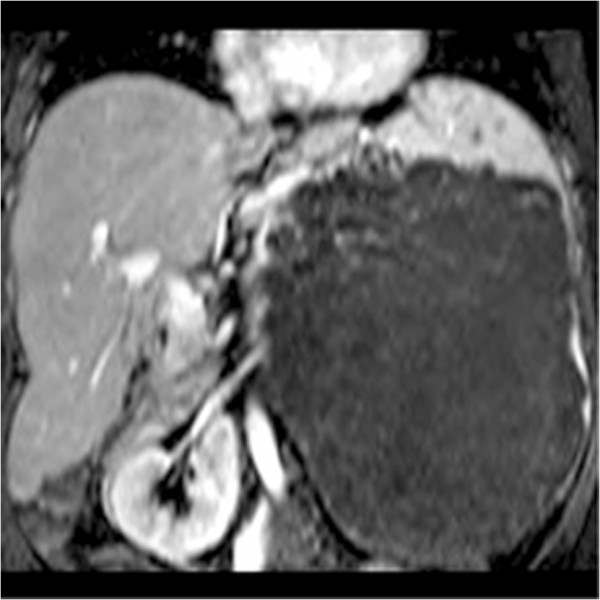
**Coronal T1-weighted plus gadolinium magnetic resonance imaging of splenic hamartoma.**

**Figure 5:**
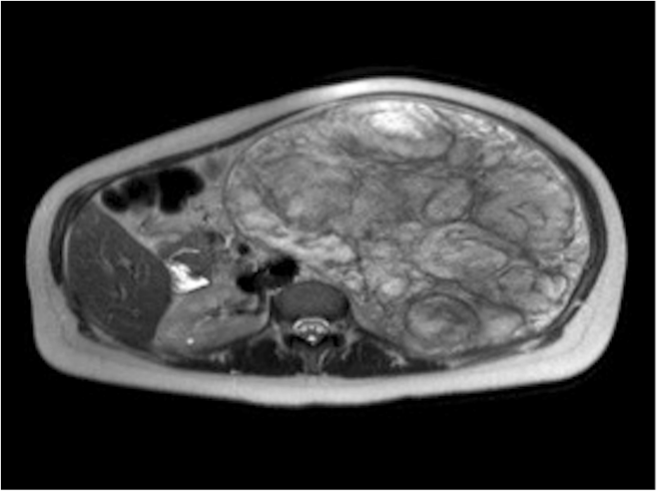
**Axial T2-weighted magnetic resonance imaging of splenic hamartoma.**

Magnetic resonance imaging (MRI) showed a high signal intensity mass on T2-weighted images, presenting intense gadolinium enhancement (Figures 4 and 5). The possibility of splenic hamartoma was proposed. This was confirmed after surgical removal of her spleen and a biopsy of the mass.

**Figure 6:**
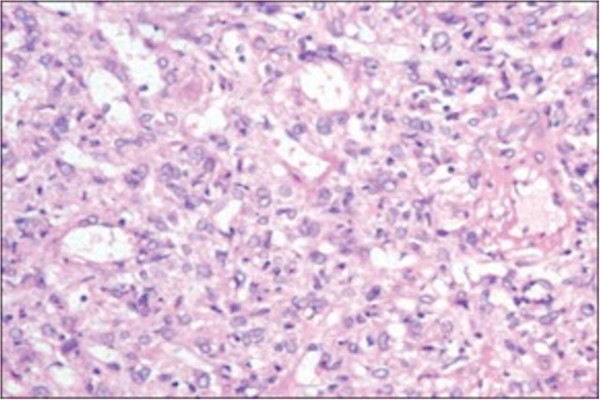
**Histological examination revealed disorganized stroma and vessels, with endothelial cells without atypia.**

Histological examination revealed disorganized red pulp-like stroma with lipocytes and disorganized vessels of variable width, lined by slightly plump endothelial cells without atypia, with or without white pulp (Figure 6).

**Figure 7:**
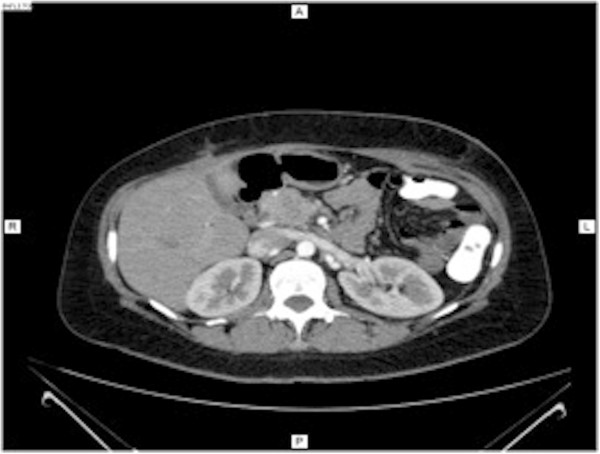
**Computed tomography imaging after surgical resection of huge splenic hamartoma.**

After surgical intervention, additional CT imaging was performed as a follow-up. This showed normal post-surgical evolution, with her other organs in normal positions and only some post-surgical lesions, such as adipose tissue thickening and fibrous remnants (Figure 7).

## Discussion

The pathogenesis of hamartomas is controversial. Some consider hamartomas to be a congenital malformation of splenic red pulp, others a neoplasm or reaction to trauma [1, 4]. The spleen is an uncommon place for primary tumor origin, so tumors located here are most commonly vascular (both benign and malignant).

Splenic hamartomas must be differentiated from other vascular tumors of the spleen depending on the cell of origin, such as hemangioma, littoral cell angioma, lymphangioma, hemangioendothelioma or angiosarcoma [4]. Solid mass-forming lesions, such as metastases and lymphoma, mycobacterial infections, and sarcoidosis must be included in the radiological differential diagnosis [5].

Hemangioma is the most common benign tumor of the spleen, arising from endothelial cells of the sinusoids and usually measuring less than 2cm [6]. Littoral cell angioma is a rare vascular tumor, arising from littoral cells of the splenic sinuses [4, 7, 8]. This rare lesion was first described in 1991 and histological findings demonstrate anastomosing vascular channels lined by cells positive to both endothelial and histiocytic markers. Lymphangioma is another rare benign spleen tumor, which manifests as a subcapsular nodule or as diffuse lymphangiomatosis in young patients. Hemangioendothelioma is a quite rare and controversial entity of the spleen, with an intermediate histology between that of a hemangioma and an angiosarcoma, with lining cells showing an intermediate degree of atypia. Finally, angiosarcoma is a malignant primary tumor of non-lymphoid origin. Irregular, anastomosing vessels, cellular atypia and invasion of adjacent organs can lead to a definitive diagnosis.

Hamartomas are quite rare lesions (about three cases in 200,000 splenectomies), which occur in any age group [1]. They seem to occur equally in male and female patients, but women seem to have larger lesions, probably due to hormonal influence. The tumor size ranges from a few millimeters up to 20cm. In our patient, the impressive finding was the enormous size (23×17×23cm) of the lesion, reaching almost the entrance of her pelvis.

Generally, hamartomas are asymptomatic. Only large lesions are able to manifest clinical symptoms, such as palpable mass, spontaneous rupture with intra-abdominal bleeding, and hypersplenism (thrombocytopenia, anemia, pancytopenia) [5, 6, 9]. This clinical symptomatology seems to occur more often in women. Most cases occur in adult patients.

Diagnosis is enhanced by imaging findings. Recently, Wang *et al.* [5] described the radiological features of hamartomas. Ultrasound findings usually include a hyperechoic mass with or without cystic areas or calcifications. Color Doppler usually shows a hypervascular mass. On CT, a hamartoma appears as a solid single or, less often, multiple masses that are well-circumscribed and encapsulated. The mass is slightly hypodense before contrast administration. After contrast injection, prolonged enhancement is achieved in a single mass, although low-density masses are observed in multiple splenic hamartomas. MRI distinguishes fibrous from non-fibrous hamartomas. MRI findings include an isointense mass in T1-weighted images and hyperintense mass in T2-weighted images [4, 8, 10, 11].

Finally, diagnosis is confirmed by histopathological examination and is enforced by immunohistopathology, with a splenic hamartoma having a cluster of differentiation 8 (CD8)-positive immunophenotype [4].

## Conclusion

Hamartoma represents a rare vascular proliferation characterized by CD8-positive immunophenotype. It is usually asymptomatic, but large tumors may present with symptoms such as hemopoetic disorders, which resolve after splenectomy. It is important for radiologists to be able to differentiate splenic hamartoma from malignant entities.

## Consent

Written informed consent was obtained from the patient for publication of this case report and accompanying images. A copy of the written consent is available for review by the Editor-in-Chief of this journal.

## Abbreviations

CT: computerized tomography
MRI: magnetic resonance imaging.
